# A Novel Homozygous Frameshift Variant in *DYM* Causing Dyggve-Melchior-Clausen Syndrome in Pakistani Patients

**DOI:** 10.3389/fped.2020.00383

**Published:** 2020-07-16

**Authors:** Nagwa E. A. Gaboon, Asia Parveen, Khaled A. Ahmad, Taghreed Shuaib, Jumana Y. Al-Aama, Lereen Abdelwehab, Amina Arif, Naveed Wasif

**Affiliations:** ^1^Faculty of Medicine, Medical Genetics Center, Ain Shams University, Cairo, Egypt; ^2^Center for Research in Molecular Medicine (CRiMM), Institute of Molecular Biology and Biotechnology (IMBB), The University of Lahore, Lahore, Pakistan; ^3^Faculty of Life Sciences, University of Central Punjab (UCP), Lahore, Pakistan; ^4^Department of Radiology, Faculty of Medicine, Ain Shams University, Cairo, Egypt; ^5^Pediatric Department, King Abdulaziz University, Jeddah, Saudi Arabia; ^6^Department of Genetic Medicine, Faculty of Medicine, King Abdulaziz University, Jeddah, Saudi Arabia; ^7^Princess Al-Jawhara Albrahim Center of Excellence in Research of Hereditary Disorders, King Abdulaziz University, Jeddah, Saudi Arabia; ^8^Faculty of Medicine, Ain Shams University, Cairo, Egypt; ^9^Institute of Human Genetics, University of Ulm and University of Ulm Medical Center, Ulm, Germany; ^10^Institute of Human Genetics, University Hospital Schleswig-Holstein, Kiel, Germany

**Keywords:** Dyggve-Melchior-Clausen syndrome, Smith-McCort dysplasia, consanguineous, Sanger sequencing, *DYM*

## Abstract

**Background:** Dyggve-Melchior-Clausen syndrome (DMC) is a skeletal dysplasia with associated defects of brain development and intelligence. The truncating pathogenic variants in *DYM* are the most frequent cause of DMC. Smith-McCort (SMC), another skeletal dysplasia, is also caused by non-synonymous *DYM* variants.

**Methods and Results:** In the current study, we examined a Pakistani consanguineous family with three affected members. Clinical features like spondyloepimetaphyseal dysplasia, indicative of characteristic skeletal abnormalities, and intellectual disability were observed. Our male patients had microcephaly and coarse facial features while the female patient did not represent microcephaly or abnormal facies, which are significant features of DMC patients. Sanger sequencing identified a novel homozygous frameshift insertion (c.95_96insT, p.W33Lfs^*^14) in *DYM*, which likely leads to nonsense-mediated decay (NMD).

**Conclusion:** The novel frameshift change verifies the fact that pathogenic variants in *DYM* are the most frequent cause of DMC.

## Background

Dyggve-Melchior-Clausen syndrome (DMC, MIM#223800) is a rare autosomal recessive skeletal dysplasia with characteristic symptoms of spondyloepimetaphyseal dysplasia (SEMD), mild to severe intellectual disability, microcephaly, coarse facial features, short stature, other skeletal abnormalities like the double-humped appearance of vertebrae and lacy iliac crests (1–5). The absence of other clinical features like corneal opacity and excretion of keratan sulfate in urine and presence of specific radiological findings differentiate it from Morquio's disease (MPS type IV, MIM 253010) (6).

DMC is caused by an alteration or complete absence of DYMECLIN (DYM) protein expression. The *in situ* hybridization studies on various embryonic stages of human development have demonstrated that the DYM expresses in human embryonic and fetal tissues during the whole human growth process (7). DYM may have a critical role in the formation and function of the Golgi apparatus and in monitoring the movements of several associated proteins and vesicles across the organelle (8). Dymeclin deficient mice (Dym^−/−^) have been reported to develop progressive skeletal deformities, suggesting skeletal phenotypes of DMC and SMC in humans (8). The particular expression of DYM has been noted in the human brain, chondrocytes, osteoblasts, and skin fibroblasts (9).

SMC is a clinical variant of DMC, sharing many skeletal phenotypes except for microcephaly and intellectual disability (9, 10). The missense variants in *DYM* reported in SMC (SMC1, MIM#607326) patients, may not affect the localization of DYM protein compared to the truncating pathogenic variants causing DMC (9). A complete absence of DYM protein in DMC patients could be suggestive of brain defects (7, 8). Mice knockout (Dym^−/−^) studied by Dupuis et al. (11), revealed the depletion of Dym protein, leading to defects in brain development and postnatal-onset microcephaly. Recently, a novel gene Ras-Related Protein RAB-33B (*RAB33B*, MIM#605950) has also been reported to cause SMC2 (MIM# 615222) (12).

We have performed a clinical and mutational diagnosis of a consanguineous Pakistani family. Clinical surveys were suggestive of DMC, and the pathogenic variant search revealed a novel homozygous frameshift insertion (c.95_96insT, p.W33Lfs^*^14) in *DYM*.

## Materials and Methods

### Enrollment of Subjects, Extraction of Blood and DNA

**Figure 1:**
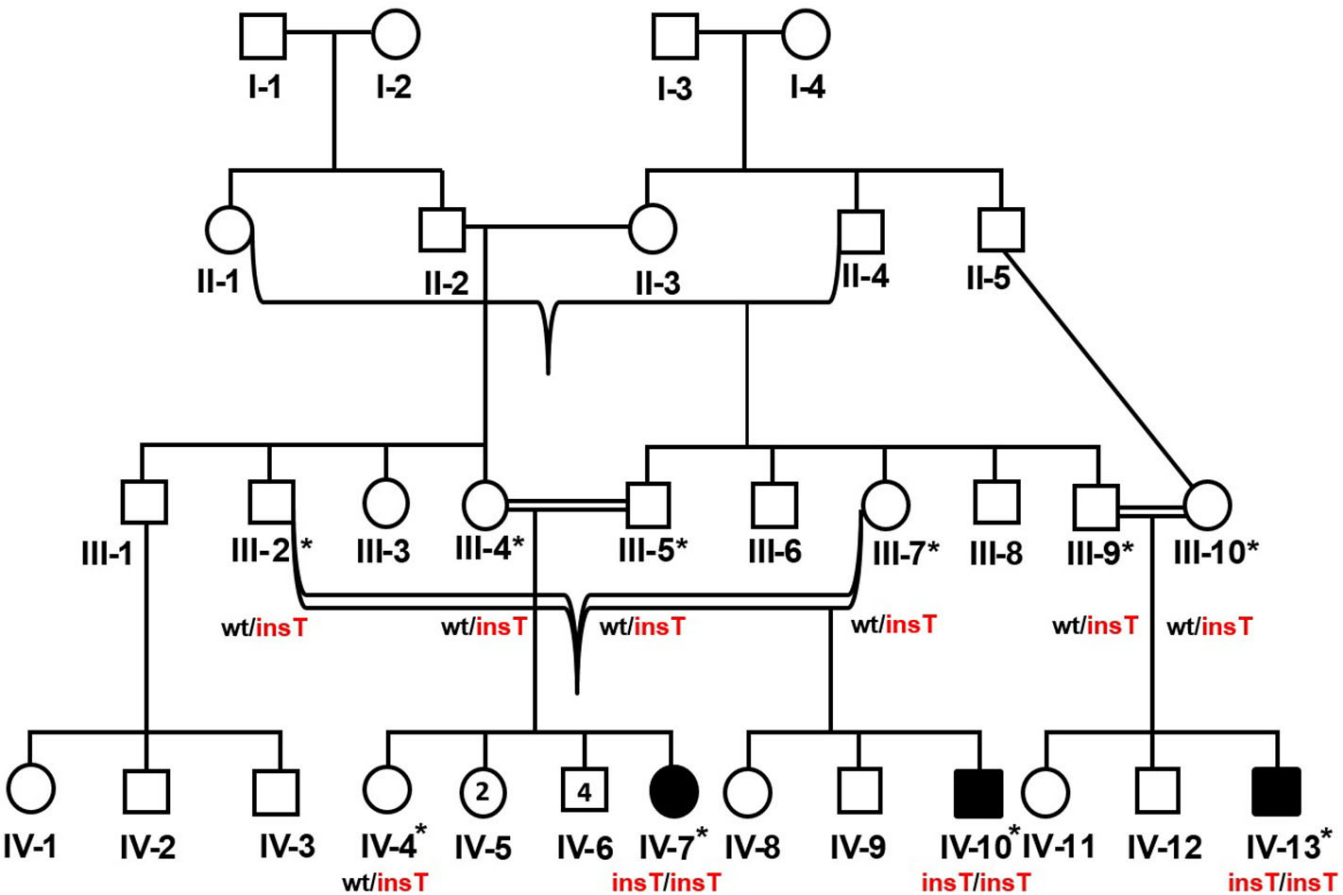
Pedigree of the DMC family in which homozygous insertion variant (c.95_96insT, p.W33Lfs*14) segregates. Available samples are labeled with asterisks, and double lines show consanguineous unions. The red-colored genotype represents the disease allele. The numbers in the symbols show the unaffected male and female siblings of IV-7.

The study included three related Pakistani patients (IV-7, IV-10, IV-13) (Figure 1). The patients were clinically evaluated at the Department of Genetic Medicine, King Abdulaziz University (KAU) Hospital, Jeddah, for a genetic consultation over skeletal deformities and intellectual disability. Written informed consent was obtained from the parents of the affected members. A pedigree construction followed detailed family history. A physical examination with particular emphasis on orthopedic findings, anthropometric measurements was done. The skeletal surveys and relevant laboratory investigations were also performed.

Peripheral blood sampling was followed by DNA extraction using the QIAamp DNA Blood Mini kit (Cat No. /ID: 51106, QIAGEN) in ten family members, including three affected and an unaffected member (IV-4, IV-7, IV-10, IV-13) and six unaffected parents (III-2, III-4, III-5, III-7, III-9, III-10). This study was approved, under a reference number 24/14, by the medical ethics and research committee of King Abdulaziz University, Jeddah, Saudi Arabia.

### DNA Sequencing

The reference sequence of *DYM* (NM_017653.6, NG_009239.2, NP_060123.3) was obtained from the University of California Santa Cruz (UCSC) genome database browser (http://genome.ucsc.edu/cgi-bin/hgGateway). The primer pairs for all 16 coding exons of *DYM* were designed using AmplifX v1.5.4 software (http://crn2m.univ-mrs.fr/pub/amplifx). The first coding exon was amplified by the following pair of primers (*DYM*-F: 5′-ggcctagccccatatttcttg-3′, melting temperature 61.9°C) (*DYM*-R: 5′-aggtggctactgagttgggtaa-3, melting temperature 60.1°C). Another forward primer (*DYM*-F: 5′-ggtagacaaagcatgatgacttttgaac-3′, melting temperature 64.2°C) was used for Sanger sequencing to perform the segregation analysis. PCR amplified the regions of interest, and the cleaning of PCR products was done by Exo-Sap protocol (https://www.thermofisher.com). The BigDye chemistry v3 was used to prepare the sequencing reaction, and DNA sequencing was performed on the ABI3730 genetic analyzer. The alignment of sequencing data against the reference genome was performed by a sequence alignment tool, BioEdit version 6.0.7 (http://www.mbio.ncsu.edu/BioEdit/bioedit.html).

The minor allele frequency (MAF) of the pathogenic variant was verified from the genome aggregation database (gnomAD, http://gnomad-old.broadinstitute.org/). A panel of 96 healthy control samples from the Pakistani population were also Sanger sequenced to exclude the possibility of a common polymorphism. The possible effect of the variant over protein stability was predicted by two online algorithms MutationTaster (http://www.mutationtaster.org/) and PROVEAN (http://provean.jcvi.org/index.php).

## Results

### Clinical Findings

#### Case-I (IV-7)

The index patient (IV-7) was a 12-years and 7 months old girl, at the time of her visit to the Department of Genetic Medicine, King Abdulaziz University (KAU) Hospital, Jeddah. She is the eighth offspring of a double first cousin Pakistani couple (41-years-old mother (III-4) and a 53-years-old father (III-5) at the time of conception). She was born full-term pregnancy with spontaneous vaginal delivery (SVD). The family history was unremarkable, and the other seven siblings (both males and females) were healthy. Her birth measures (length, weight, occipitofrontal circumference) were within the normal range in reference to her gestational age.

**Figure 2:**
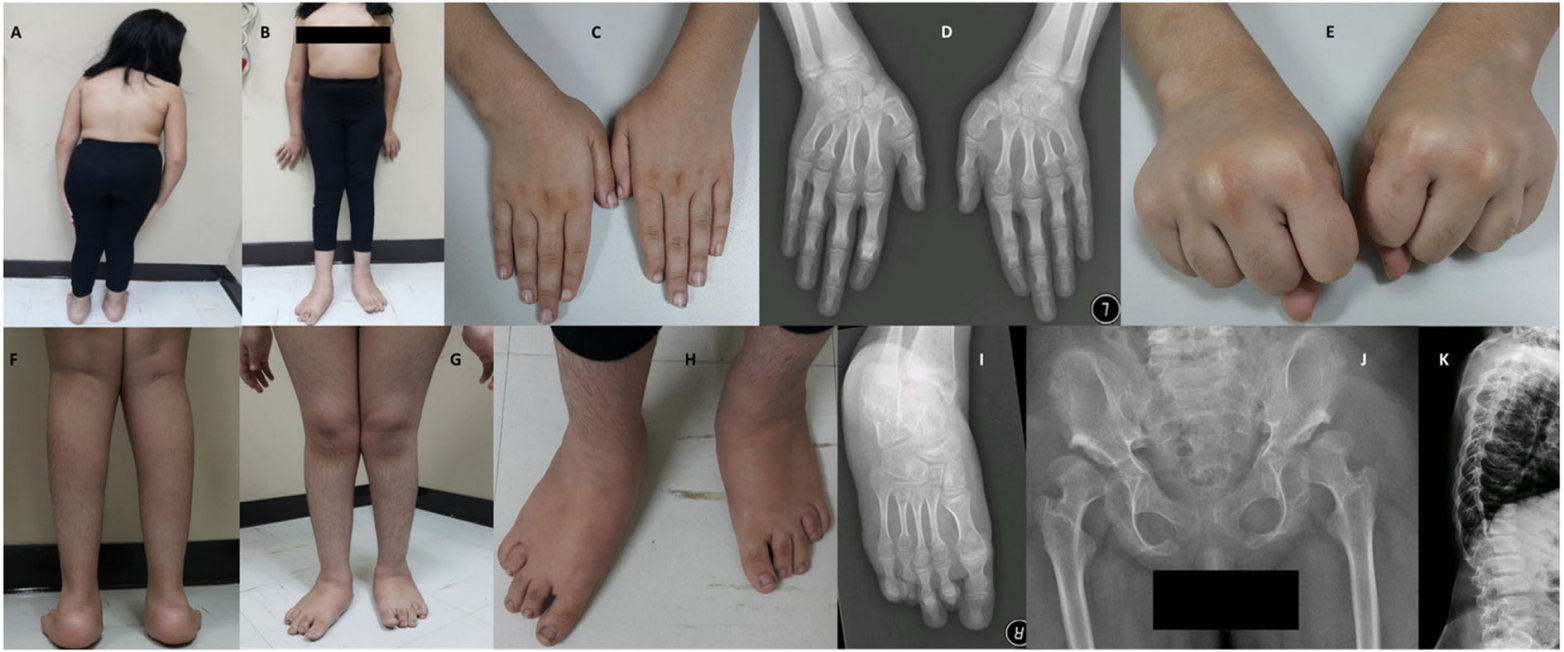
Photos and X-rays of Case-I (IV-7) are showing short trunk, coxavara, mild genu valgum **(A,B)** short first, fourth and fifth fingers and metacarpals **(C,D)** and absent fourth and fifth knuckles during fist formation **(E)**, prominent heels, pes planus, brachydactyly of toes more prominent 4th and 5th with short metatarsals **(F–I)**, A-P hips X-rays shows bilateral shallow saucerized acetabulum with abnormally flat and enlarged femoral head and narrow femoral neck, lace-like small iliac wings and bilateral coxa valga **(J)**. X-rays of spines show double hump appearance with antero-inferior beaking of the vertebral bodies **(K)**.

The patient had early healthy motor development, speech delay, and subnormal mentality. At 3-years of age, she was observed to have progressive skeletal changes (Figures 2A,B), with a waddling gait, difficulty to rise-up after sitting down and urine incontinence. History of convulsion or neurological manifestations were not reported.

During the clinical examination, she had good eye contact with no hyperactivity or anxiety. She could understand and obey simple commands and could pronounce a few unclear words. She could walk under assistance. On assessment at the age of 12-years and 7 months, she had short stature (123 cm,−4.4 SD), lower segment (LS) 67 cm, upper to lower segment ratio (US/LS) 0.83 (standard ~0.9), skull circumference was on the 26th percentile (53.5 cm, −0.63 SD) and weight was on a fifth percentile (32 kg). She had low hairline, broad chest, broad elbow, and broad wrist joints (Figures 2B–E). Short and broad hands with brachydactyly of first, fourth, and fifth fingers, with absent fourth and fifth knuckles during fist formation (Figures 2C–E). The lower limbs showed bilateral coxa valga, pes plans, prominent heels, bilateral brachydactyly of fourth and fifth toes (more in fourth), bilateral camptodactyly of second and third toes, and bilateral wide sandal gap (Figures 2F–I). The radiological findings coincided with the clinical reports; the radiological analysis revealed the abnormal development of hip joints and bones (Figure 2J). X-rays of spines revealed a double hump appearance with antero-inferior beaking of the vertebral bodies (Figure 2K). The patient had an intellectual disability (IQ = 60) and generalized familial hypertrichosis. No corneal clouding, hearing deficit, or involvement of other systems were identified.

#### Case-II (IV-10)

A 13-year-old male patient (IV-10) was born to healthy double first cousin parents following an uncomplicated pregnancy and vaginal delivery. At the time of his conception, father (III-2) and the mother (III-7) were 56- and 40-years of age. There was a history of two neonatal deaths (a male and a female) with unknown cause, two unaffected siblings (IV-8, IV-9), and two affected cousins (IV-7, IV-13). The patient (IV-10) had an early global developmental delay, with progressive skeletal changes and abnormal gait, observed at the age of two.

**Figure 3:**
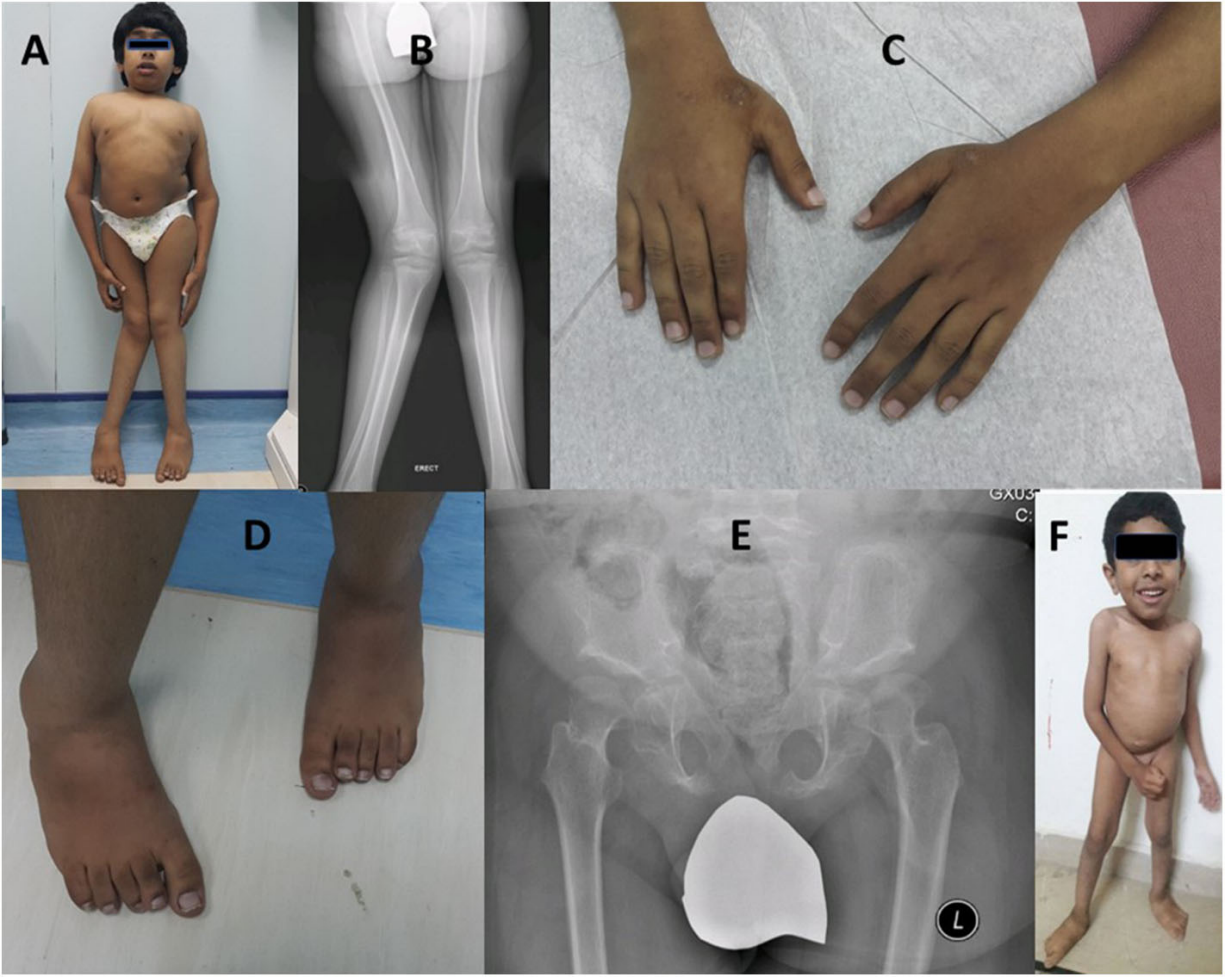
Case-II (IV-10) presenting short trunk, genu valgum more appreciated on the right side **(A,B)**, brachydactyly of fingers **(C)**, short toes with right second toe overriding the first **(C,D)**. Radiological diagram of hip shows shallow acetabulum with abnormally flat and enlarged femoral head and narrow femoral neck, lace-like small iliac wings, and bilateral coxa valga **(B,E)**. Case-III is a 4-year-old boy (IV-13) showing short trunk, broad chest, and pes planus **(F)**.

He was short 115 cm (−5.6 SD) and microcephalic, with a head circumference of 48 cm (−4.16 SD). His weight was on the 10th percentile (36 kg), U/S 0.77 (normal ~0.9), coarse facial features, low hairline, and prominent lips. He had a short trunk, short chest, broad elbow, and wrist joints, and asymmetric genu valgum more appreciated on the right side (Figures 3A–C). He showed short, broad hands and feet with brachydactyly of all fingers and toes equally, unlike his cousin (case-I, IV-7), pes planus, prominent heels, and generalized hypertrichosis (Figures 3C,D). Radiological analysis of the hip joint revealed shallow acetabulum with abnormally flat and enlarged femoral head and narrow femoral neck, lace-like small iliac wings, and bilateral coxa valga (Figure 3E). Intellectual disability with an IQ = 41 was recorded. Abnormalities of other body organs, visual and hearing impairment were not observed.

#### Case-III (IV-13)

A 4-year-old male patient (IV-13), third offspring of the first cousin parents (III-9, III-10), belonging to the same family tree, had major complaints of not gaining height, delay in developmental milestones, and intellectual disability. On physical examination, mild short trunk, broad chest, pes plans, and microcephaly (Figure 3F) were observed. He had a height of 94 cm (−2.25 SD), lower segment 49 cm, U/L segment 0.92 (normal ~1.2), weight 11 kg (<3rd centile), and skull circumference 43 cm/−4.86 SD.

### Investigations

Karyotyping, erythrocyte sedimentation rate (ESR), C-reactive protein, ANA (antinuclear antibodies), complete blood count, coagulation profile, GAG (glycosaminoglycans) in the urine, urea and electrolytes, thyroid function tests, parathyroid hormone, serum calcium, phosphorus, and alkaline phosphatase, liver enzymes were all within normal ranges as compared to the reference values, in the three affected members (IV-7, IV-10, IV-13). The echocardiogram and abdominal ultrasound were unremarkable.

### Screening for a Pathogenic Variant

**Figure 4:**
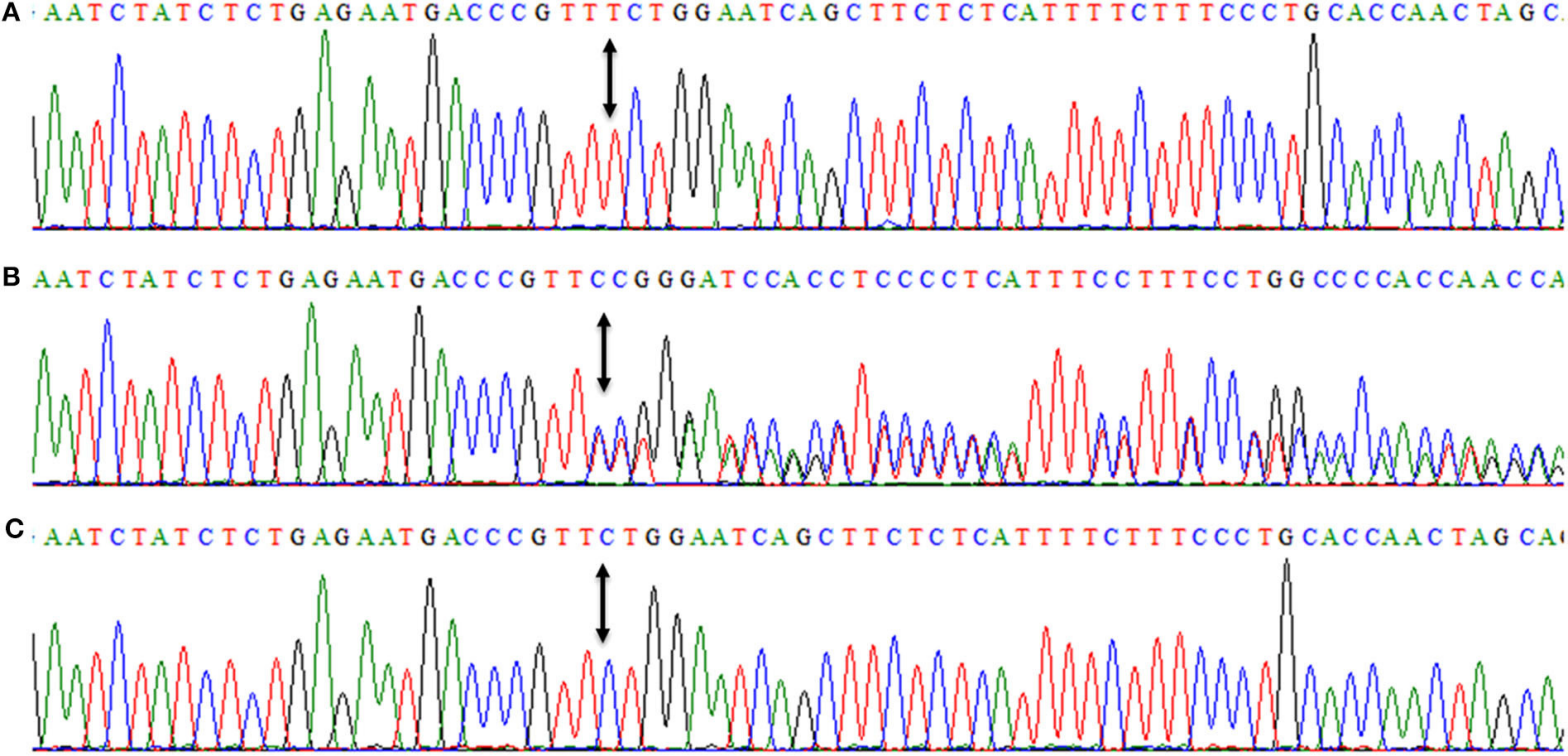
Nucleotide sequence of first coding exon of *DYM* showing homozygous frameshift variant (c.95_96insT, p.W33Lfs*14) in an affected member (IV-7) **(A)**, while **(B)** is showing the DNA sequence of a heterozygous carrier (III-4). **(C)** is showing a DNA sequence of a control individual. Arrows between the sequences are showing the position of the genetic alteration. The above nucleotide sequences are obtained from same forward primer (DYM-F: 5′-ggtagacaaagcatgatgacttttgaac-3′, melting temperature 64.2°C), used for the Sanger sequencing reaction.

DNAs of all three affected members (IV-7, IV-10, IV-13) were subjected to PCR amplification of all 16 coding exons of *DYM*. DNA sequencing revealed a novel insertion variant (c.95_96insT, p.W33Lfs^*^14) in the first coding exon (Figure 4). This variant was present in a heterozygous state in unaffected family members (III-2, III-4, III-5, III-7, III-9, III-10, IV-4), confirming its segregation within the family. The variant was not present in 96 healthy controls of Pakistani population. To date, MAF of this variant is not cited in gnomAD. MutationTaster and PROVEAN (pathogenicity score: −8.10) have predicted this novel alteration (c.95_96insT) as a frameshift variant causing a deleterious effect on protein structure. Due to the unavailability of the crystal structure of DYM and reliable templates via PSI-BLAST (https://www.ebi.ac.uk/Tools/sss/psiblast/) search, the structural studies to investigate the impact of the pathogenic variant were not carried out.

## Discussion

Our study includes three related DMC patients (IV-7, IV-10, IV-13). DMC includes various phenotypes like short stature, short trunk, and intellectual disability. Additional symptoms of DMC are microcephaly, coarse facial feature, rhizomelia, and bone deformity (5, 11).

During the clinical surveys of our patients, including body examination, and radiological investigations, a clinical feature of rhizomelic shortening was not established in all cases, and case-I (IV-7) did not manifest microcephaly. These are the prominent features of DMC patients, as described in several previous studies (4, 5, 10, 13). The manifestation of mild to severe intellectual disability in all three affected members and microcephaly in male patients, helped us to categorize them as DMC patients instead of SMC patients. The absence of deafness and corneal opacity excluded the chances of Morquio's disease (A and B type), which is caused by the deficiency of N-acetylgalactosamine-6-sulfatase and beta-galactosidase. The excretion of these enzymes in urine was within the normal range in our patients.

Coarse facial features were prominent in case-II and case-III (IV-10, IV-13) in comparison to the female patient (case-I, IV-7). Aglan et al. (4) have reported similar findings in their patients; in which coarse facial feature was more severe in their male cases than the affected females. On the other hand, the female index (case-I) showed significant bilateral shortening of first, fourth, and fifth fingers, and fourth and fifth toes. To the best of our knowledge, this is a distinct clinical feature not described in already reported DMC cases.

DNA sequencing has identified a novel homozygous frameshift variant (c.95_96insT, p.W33Lfs^*^14) in *DYM* in affected members. *DYM* variants have already been described in the literature as the leading cause of the DMC phenotype.

*DYM* has been mapped at chromosome 18q21.1, encoding a 669 amino acids protein. DYMECLIN (DYM) plays an important role in the regulation of the organization of the Golgi apparatus and morphogenesis of bones (14). To date, HGMD Professional 2018.4 (http://www.hgmd.cf.ac.uk/ac/gene.php?gene=DYM) has a registry of forty-two pathogenic variants [missense/non-sense (17 variants), splicing substitutions (11 variants), deletions (10 variants), and insertions/duplications (4 variants)] in DYM, causing DMC, SMC1, intellectual disability, and other dysmorphic phenotypes. Only twenty-seven *DYM* variants have been reported to cause DMC (HGMD Professional 2018.4) in patients from various countries around the globe, like Lebanon, Morocco, Tunisia, Pakistan, and India (3, 9).

DYM protein is a non-anchored Golgi protein that shuttles freely from one cellular compartment to another (7, 8, 14). In a study conducted by Denais et al. (14), it has been described that DYM may have an association with some structural Golgi proteins called soluble NSF [N-ethylmaleimide (NEM)-sensitive fusion protein] attachment protein receptors (SNAREs) and Golgi reassembly stacking proteins (GRASPs) (15–17). These structural proteins play their role in the formation of Golgi ribbons and in developing interactions between these ribbons (14). In the same study, it is illustrated that genetic alterations in genes, encoding regulatory protein for secretion and maintenance of Golgi structure, may affect the development of skeletal muscles (14). In recently published review articles, Passemard et al. (18) and Rasika et al. (19)have classified DMC syndrome as a part of a newly described group of Golgipathies, characterized by skeletal and intellectual defects and caused by disease-causing variants in several Golgi proteins, including DYM. Electron microscopic analysis of chondrocytes and cutaneous cells of affected individuals revealed the dilated cisternae of the rough endoplasmic reticulum (9, 20), which may suggest DMC as a storage disease (2). This enlargement of rough endoplasmic reticulum may lead to inadequate extracellular matrix formation, which can be a leading cause of impaired bone morphogenesis in DMC and SMC patients (14).

In the current study, we have identified the fifth homozygous insertion variant (c.95_96insT, p.W33Lfs^*^14) in *DYM* in a consanguineous family of Pakistani origin. According to MutationTaster, this frameshift variant may lead to NMD, likely to result in the complete absence of DYM protein. NMD is a conserved mammalian surveillance system which destroys the imperfect messages, generated by premature stop codons (21). Dimitrov et al. (7), have explained that the pathogenic variations in *DYM* with premature stop codons (PTC), either in the form of non-sense, splice site, deletions, or insertions, may lead to mislocalization and/or degradation of DYM protein in DMC patients.

The present study increases the mutation spectrum of *DYM* and confirms the notion that the deleterious variants causing PTC are the leading cause of human DMC.

## Data Availability Statement

The datasets generated for this study can be found in the ClinVar, SUB6990858, https://submit.ncbi.nlm.nih.gov/subs/variation_clinvar/SUB6990858/ (accession number SCV001161768).

## Ethics Statement

This study was approved, under a reference number 24/14, by the medical ethics and research committee of King Abdulaziz University, Jeddah, Saudi Arabia. Written informed consent to participate in this study was provided by the participants' legal guardian/next of kin. Written informed consent was obtained from the individual(s), and minor(s)' legal guardian/next of kin, for the publication of any potentially identifiable images or data included in this article.

## Author Contributions

NG, KA, TS, JA-A, and LA have enrolled the patients and contributed to the radiological analysis, clinical diagnoses, and report writing. NW contributed to Sanger Sequencing. NG and AP have written the initial draft of the manuscript. NW, AA, and NG have critically reviewed and finalized the manuscript. All authors read and approved the final draft.

## Conflict of Interest

The authors declare that the research was conducted in the absence of any commercial or financial relationships that could be construed as a potential conflict of interest.
